# Steering herds away from dangers in dynamic environments

**DOI:** 10.1098/rsos.230015

**Published:** 2023-05-24

**Authors:** Stef Van Havermaet, Pieter Simoens, Tim Landgraf, Yara Khaluf

**Affiliations:** ^1^ Department of Information Technology, University of Ghent—imec, Technologiepark 126, 9052 Ghent, Belgium; ^2^ Department of Mathematics and Computer Science, Freie Universität Berlin, Arnimallee 7, 14195 Berlin, Germany; ^3^ Department of Social Sciences, Wageningen University and Research, Hollandseweg 1, 6706KN Wageningen, The Netherlands

**Keywords:** shepherding, collective motion, multi-agent system, decentralized decision-making

## Abstract

Shepherding, the task of guiding a herd of autonomous individuals in a desired direction, is an essential skill to herd animals, enable crowd control and rescue from danger. Equipping robots with the capability of shepherding would allow performing such tasks with increased efficiency and reduced labour costs. So far, only single-robot or centralized multi-robot solutions have been proposed. The former is unable to observe dangers at any place surrounding the herd, and the latter does not generalize to unconstrained environments. Therefore, we propose a decentralized control algorithm for multi-robot shepherding, where the robots maintain a caging pattern around the herd to detect potential nearby dangers. When danger is detected, part of the robot swarm positions itself in order to repel the herd towards a safer region. We study the performance of our algorithm for different collective motion models of the herd. We task the robots to shepherd a herd to safety in two dynamic scenarios: (i) to avoid dangerous patches appearing over time and (ii) to remain inside a safe circular enclosure. Simulations show that the robots are always successful in shepherding when the herd remains cohesive, and enough robots are deployed.

## 1. Introduction

Agents capable of guiding a group of other autonomous agents are essential to a wide range of applications [1]. Robotic agents can be used to herd animals such as sheep [2,3], cattle [4] and ducks [5], to enable crowd control [6], to keep birds away from aircraft [7,8], and to bring people to safety [9]. In the literature, this type of guidance is studied under the name of shepherding, as it is inspired by dogs herding sheep to a desired location [10]. Sheepdogs are particularly skilled at shepherding, with a single dog capable of herding more than 80 sheep [11]. These dogs have learned to exploit the sheep’s collective behaviour of aggregating and together escaping from a threat [12]. This behaviour is widely accepted as an example of the selfish herd theory [13]. Supporting evidence for this theory has been found for other animal species. For instance, fish move together in schools to reduce predation risk [14].

The individual mechanisms that animals apply to establish and maintain aggregated formations have been studied broadly in research. Various models of collective motion have been proposed in both theoretical [15–18] and empirical [19,20] studies over the past decades. A recurring feature in these animal models is that the individual behaviour results from a few simple rules that take as input the motion of a relatively small set of neighbours [21,22]. Reynolds [23] proposed one of the earliest models for the flocking of birds based on three distinct rules: (i) to avoid collision with close neighbours, (ii) to move in the same direction and at the same speed as others and (iii) to remain as a cohesive group. These interactions have been proposed as the founding blocks for the underlying behavioural mechanisms in fish schools [24], mammal herds [25], pedestrian crowds [26] and other vertebrates [27]. Most notably, Couzin *et al.* [28] showed how the information from only a few informed individuals can propagate to the entire collective system. When some individuals gain information about the location of a danger and consequently change their directions towards a safer region, these changes propagate in the entire group. Hence, the actions of a single sheepdog can influence the collective motion of the entire herd of sheep, even though only a small part of the herd directly observes the sheepdog.

Robotic agents, visually styled to trigger an aversive response of the herd, can rely on the same shepherding mechanisms as natural perceived threats, like dogs [29]. This corresponds to the robot computing the optimal motion control vector based on force vectors representing the interactions among herd members, and the interaction between the herd and a shepherd. In the classic shepherding problem, the shepherds are tasked with guiding the herd to a certain goal location, that is automatically known to the shepherds as prior information. In this paper, however, we study the problem setting where this assumption is relaxed. The shepherds must actively determine the goal location based on local information and communication. Such shepherds could be beneficial in protecting animal herds from unforeseen dangers in dynamic environments, by guiding them to a safer location. To mimic real-life use cases, we thus consider that the robotic shepherds can only obtain and communicate information in a local radius, and they have no prior information about the dangers. Once a danger has been detected, the shepherds are tasked with guiding the herd away from this danger.

We propose a solution to the previously described problem where the herd is consistently surrounded by multiple shepherds. We also refer to this formation as *caging* the herd, as the shepherds are evenly spread out across the contour of the herd. As such, the shepherds can detect any nearby danger approaching the herd. Additionally, with a sufficient number of shepherds, the task of steering the herd can be allocated to the shepherds already present in the appropriate region based on the location of the detected danger. Similar to other shepherding research works, we model a member of the herd to move in the opposite direction of a shepherd when the relative distance is lower than a certain threshold. The shepherds positioned between the herd and a danger will therefore move close enough to the herd, which should trigger the herd to change direction. When the herd is not approaching a danger, the shepherds remain far away enough, which allows the herd to continue their natural behaviour (e.g. foraging).

Long *et al.* [1] stated several challenges in their literature review of robotic shepherding. We believe our approach advances the state-of-the-art of shepherding with respect to three of these challenges. Firstly, the models they have reviewed are not easily transferable to dynamic environments. In our work, the proposed algorithm is demonstrated in two dynamic environments: (i) dangerous patches appear nearby the herd at a certain probability and (ii) a circular safe zone containing the herd decreases in size over time. Secondly, a shepherd should be able to dynamically adapt the distance threshold where the herd tends to move away from the shepherd, while being limited in energy consumption and computation time. Therefore, we design the artificial intelligence of the shepherds through a set of control rules, where the threshold is explicitly incorporated in the computations. Thirdly, Long *et al*. argue that practical applications of shepherding require robustness with regard to the failure of robotic agents. Hence, we propose a decentralized, multi-agent shepherding algorithm where each shepherd gathers information from local radius-based observation and is able to communicate information to nearby neighbours. Contrary to the majority of the literature so far [30], no central unit providing commands or global information is available to the shepherds in our work.

We apply our approach specifically to the shepherding of fish, motivated by the biomimetic robotics state-of-the-art of fish-like robots [31] and the potential environmental impact. Although fish are an important part of our natural environment [32], they are increasingly faced with dangers such as illegal fishing [33], pollution [34] and invasive species [35]. The efficiency of the proposed algorithm is demonstrated through simulation, where four different models of collective motion are used to simulate the fish behaviour. One of these models was proposed by Couzin *et al.* [24] and has been shown by multiple empirical studies to capture the key features of fish behaviour such as nearest-neighbour distance, polarization, group speed and turning rate [20,36–40]. More specifically, we simulate the herd as guppies who live in shallow waters, which applies to our proposed algorithm designed for two-dimensional environments [41].

The rest of this paper is organized as follows. Section 2 briefly discusses the most prominent research works on the topic of shepherding and the studies that have inspired our proposed algorithm. In §3, we formulate the three main parts of the problem scenario: (i) the four considered models of collective motion to simulate the herd, (ii) the definition of caging and (iii) the descriptions of the shepherding tasks. Next, in §4, an algorithm for establishing and maintaining a caging formation is proposed, and afterwards one for shepherding (while caging) the herd in the presence of danger. Results of the respective main parts of the problem scenario are discussed in §5. Finally, we conclude the paper in §6.

## 2. Related work

The Robot Sheepdog Project [42] was one of the first research projects to develop a robot capable of solving the classic shepherding problem, based on a computation of force vectors that represent the inter-individual rules proposed by Reynolds [23]. A single robot used a ceiling-mounted camera to track a flock of ducks and manipulate their movement. In order to direct the flock in the right direction, the robot positions itself on the opposite side of the flock to the goal. When the robot is correctly aligned with respect to the goal and the flock, it advances towards the flock and hence the flock moves towards the goal. However, the robot and the ducks were placed in a circular enclosure where the goal location was always placed at the edge of the enclosing circle. The robot shepherded the ducks to move along the edge until the goal location was reached.

Some follow-up works have adapted the aforementioned algorithm in order to shepherd in unconstrained environments. Strömborn *et al.* developed a single-agent shepherding algorithm based on force vectors, where the shepherd switches between collecting dispersed herd members and steering the cohesive herd [43]. This results in a side-to-side motion of the shepherding agent behind the herd, which mimics the behaviour of real sheepdogs. Miki and Nakamura developed a similar algorithm, where the shepherd adaptively switches between collecting and steering the herd [44], but included a notion of cooperation between multiple shepherds who avoid overlapping. Their experiments showed that two shepherds are more efficient in guiding the herd than only one. Other studies corroborated the finding that single-agent solutions are limited to smaller herd sizes [45], and thus multiple shepherds can control large herds more efficiently than a single shepherd [46].

However, most shepherding control approaches assume that the shepherds have global knowledge of the positions of every individual in the environment [1]. Applying robotic shepherding to any environment means that the robots can only rely on local information gathered by sensors with limited range [47–49]. Tsunado *et al*. proposed an algorithm where a single shepherd constantly aims to repel the furthest herd member from the goal location towards it, using only information collected via a simulated local camera [30]. This algorithm was shown to be successful in simulations, while the algorithm proposed by Strömborn *et al.* [43] mostly failed to guide the herd to the goal when only local information is available to the shepherd. The challenge of moving a herd to a goal with multiple shepherds, using only local information, was tackled by Lee & Kim [50]. Shepherds coordinate to aggregate one cohesive herd, where some steer wandering members towards the main herd, while others focus on keeping the main herd at its current position. Only when the herd is cohesive enough, each shepherd repels the closest herd member towards the goal location.

A simpler but effective algorithm was presented by Miki *et al.* [44], where the shepherd moves in a circular motion behind a member of the herd (relative to the guidance direction) and then comes closer to repulse the herd member. Each herd member followed the traditional Reynolds flocking algorithm in their work, where stochastic behaviour is considered. Experiments were conducted with one shepherd steering a herd of 25 individuals, and two shepherds cooperating to steer 30 individuals. This steering algorithm is similar to the one we present in this paper, where the shepherding robots have to cooperate and only obtain partial knowledge of the environment through local sensing.

As discussed beforehand, in the problem setting that we address, the shepherds are required to maintain a *caging* formation while guiding the herd away from dangers. Varava *et al.* [51] refer to the problem of steering a herd while maintaining a caging formation as *herding by caging*. They proposed a centralized RRT-based (rapidly exploring random tree) algorithm using computational topology techniques to verify the caging formations. However, their approach assumes global information and an initial correctly caged formation. Furthermore, they did not consider the natural motion of the herd in the absence of robots, and their simulations suggest that small increases in the number of robots result in a significant decrease in the algorithm’s performance.

Our approach for shepherds to establish and maintain a caging formation is inspired by the simple behavioural rules of wolf-pack hunting strategies [52]. For a stationary single prey, the wolves become arranged in a stable configuration of a regular polygon, which we refer to as a caging formation. While the authors do not provide any formulas, they state that wolves hunt following only two simple decentralized rules: (i) the wolf moves towards the prey until a safe distance threshold is reached and (ii) when close enough to the prey, the wolf moves away from the other wolves that are close to the safe distance to the prey. To successfully follow these rules, the wolves do not need to rely on direct communication, nor is there a role of leader needed in the group. Hence, these rules are applicable to our problem scenario, where the shepherds operate in a decentralized system.

## 3. Problem formulation

Let A and H denote the respective sets of robotic agents and herd members. The state of the discrete-time system at time *t* is defined by the position $p_{i}(t)\in R^{2}$ and orientation *θ*_*i*_(*t*) ∈ [0, 2*π*) of each individual i∈A∪H. At every time step, an agent first rotates with angular velocity *w* to its desired orientation $\hat{\theta }$ that is computed based on local information. The agent stops rotating once $\hat{\theta }$ is reached, or when the time interval has passed. In the remainder of the time interval, the agent moves straight forward at linear velocity *v*. At every time step, Gaussian noise *σ* is added to the orientation of each individual.

### 3.1. Collective motion models

**Figure 1.**
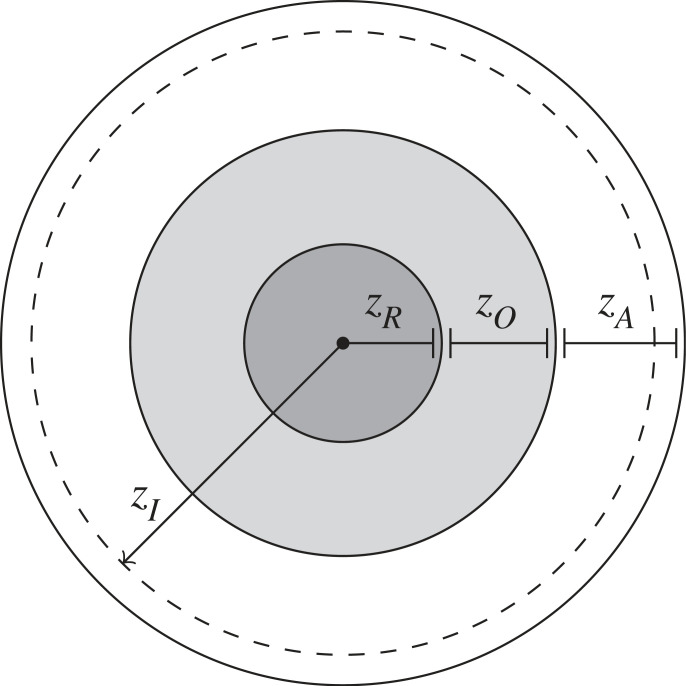
Behavioural zones of the collective motion models.

Each herd member updates its direction of motion based on three concentric non-overlapping zones (figure 1), containing distinct subsets of neighbours. Each zone corresponds to a type of interaction: (i) repulsion from others inside the disc with radius *z*_*R*_, to establish a minimum inter-individual distance, (ii) alignment of orientation with others inside the annulus with width *z*_*O*_, to all move in the same direction, and (iii) attraction to others inside the annulus with width *z*_*A*_, to remain as one cohesive group.

**Figure 2.**
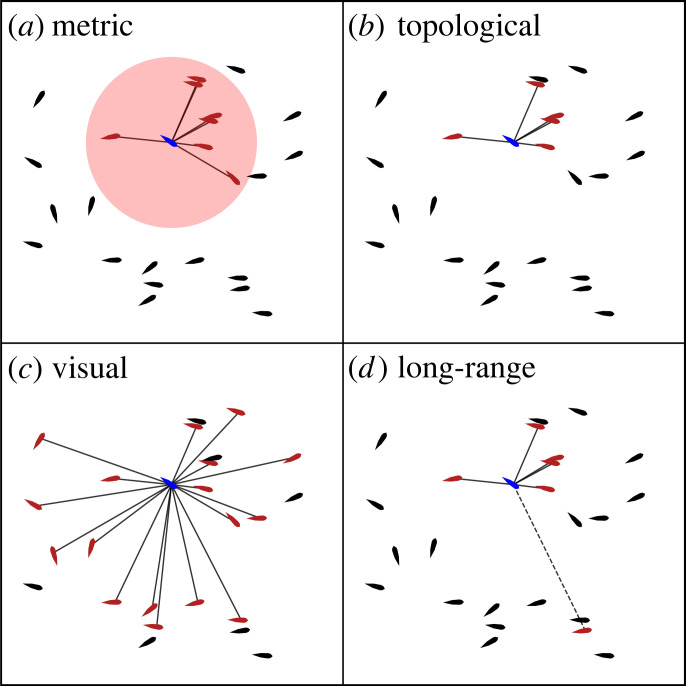
The set of neighbours (red) is illustrated in regards to a focal individual (blue) for the different models of collective motion. (*a*) Metric: all individuals within a certain radius. (*b*) Topological: only the *k* nearest neighbours (*k* = 5 in this example). (*c*) Visual: all individuals that are visually observable, i.e. not obstructed by any other neighbour. (*d*) Long-range: in addition to topologically selecting neighbours (solid line with *k* = 5), there are *λ*_*i*_ randomly selected neighbours (dashed line with *λ*_*i*_ = 1).

In this paper, we consider four different models of collective motion that have been empirically studied in fish schools [53]: metric, topological, visual reconstruction and long-range (figure 2). These models differ from one another in the way that neighbours $N_{i}$ of each individual *i* are selected. Using the metric model, the set of neighbours consists of all other individuals within the interaction radius *z* = *z*_*R*_ + *z*_*O*_ + *z*_*A*_. In the topological model, $N_{i}$ only contains the *k* nearest neighbours. Visual reconstruction only selects other individuals that can be visually perceived as neighbours, which means no other individual obstructs the focal individual *i* from observing the peripheries of a neighbour. Finally, in the long-range model, short-range interactions are considered by selecting *k* nearest neighbours (i.e. topologically), while long-range interactions are defined by randomly selecting *λ*_*i*_ neighbours from the remaining members of the herd. The number of long-range neighbours *λ*_*i*_ is sampled from a Poisson distribution with average *λ* as parameter of the model.

To model the aversive behaviour of a herd individual *i* interacting with a robotic agent, an additional disc with radius *z*_*I*_ surrounding *i* is introduced. A herd individual wants to move in the opposite direction (i.e. repulsion) of any robot present in this zone. Later in this paper, we will introduce two different scenarios for shepherding, where D is a set containing (i) the centres of dangerous patches or (ii) the centre of a safe circular enclosure. The herd interacts with these dangers likewise to robotic agents.

Let $N_{i}^{R}$, $N_{i}^{O}$ and $N_{i}^{A}$ then denote the distinct subsets of neighbours by separating $N_{i}$ based on the repulsion, orientation and attraction zones, respectively. Thus, each neighbour is only assigned to one of the subsets: $N_{i}^{R}\cap N_{i}^{O}\cap N_{i}^{A}=\emptyset$. Let $A_{i}$ and $D_{i}$ denote the respective subsets of robotic agents and dangerous patches located in a radius of *z*_*I*_ around *p*_*i*_. In the scenario of the enclosure, $D_{i}$ contains the centre of the safe disc if and only if the individual *i* is located outside of the disc.

Let us define the relative position of individual *j* from *i* as *r*_*ij*_(*t*) = *p*_*j*_(*t*) − *p*_*i*_(*t*). Furthermore, let *q*_*i*_ be the motion vector of an individual *i*, which is computed as follows:

$$\begin{array}{rl} q_{i}(t) & =-\alpha_{R}\sum_{\;j\in N_{i}^{R}}\frac{r_{ij}}{\left\|r_{ij}\right\|}+\alpha_{O}\sum_{\;j\in N_{i}^{O}}\frac{q_{j}}{\left\|q_{j}\right\|}+\alpha_{A}\sum_{\;j\in N_{i}^{A}}\frac{r_{ij}}{\left\|r_{ij}\right\|}-\alpha_{I}\sum_{\;j\in A_{i}\cup D_{i}}\frac{r_{ij}}{\left\|r_{ij}\right\|}, \end{array}$$

with weights *α*_*R*_ ≥ 0, *α*_*O*_ ≥ 0, *α*_*A*_ ≥ 0 and *α*_*I*_ ≥ 0 of repulsion, orientation, attraction and animal–robot interactions, respectively.

### 3.2. Caging

A caging formation can be constructed by the robotic agents based on the repulsive animal–robot threshold *z*_*I*_. When two robotic agents are at a distance lower than 2*z*_*I*_ from each other, they exert a combined repulsive force on the herd which prevents them from intersecting the path between those agents. In other words, caging is equivalent to a closed chain formation where the distance between consecutive agents satisfies the upper bound of 2*z*_*I*_.

In order to measure whether the agents established an appropriate caging formation, we see if a polygon can be constructed from the edges between agents where the length is shorter than 2*z*_*I*_. Following [51], we then define the herd to be successfully caged, if and only if, the polygon is closed and the entire herd is located in the interior of the polygon.

### 3.3. Shepherding

The agents’ objective is to prevent any herd member from entering dangerous areas. When a danger is detected to be approaching the herd, the agents are tasked to preemptively steer the herd away. In the absence of dangers, the agents should remain at a minimum distance of *z*_*I*_ from the herd to avoid any unnecessary stress induced on the herd. The agents have no prior knowledge about the positions and the movements of the dangers, which can only be locally observed.

In this paper, we consider two different shepherding tasks based on common scenarios in which animal herds would benefit from safety interventions. In the first task, a stationary dangerous patch appears in front of the herd at a certain probability. This task resembles several use cases of different dangers such as pollution (e.g. fish near oil leaks) and poachers. In the second task, the robots are expected to ensure that the herd remains in a given safe zone. This task is similar to deploying virtual fences. The use of mobile robots also allows for the safe zone to be dynamically changed, which is useful for caretaking of animals. For example, it allows to navigate the herd between nests and optimize grazing patterns.

We model the task environment by a time-variant potential function *f* that reaches a local minimum in the mean direction opposite of all dangers (assuming that each danger is equally important to avoid). Consequently, the herd should move in the direction of the potential gradient ∇f. In the first task, agent *i* computes the gradient based on the relative positions of the observable dangerous patches $D_{i}$ as follows: $\nabla f\;(p_{i})=-\sum_{d\in D_{i}}((\;p_{d}-p_{i})/\left\|p_{d}-p_{i}\right\|)$. In the case where the agent does not detect any dangers, the gradient is undefined. In the second task, the potential gradient is defined as $\nabla f\;(p_{i})=(\;p_{e}-p_{i})/\left\|p_{e}-p_{i}\right\|$ with *p*_*e*_ the centre of a circular safe zone. In the case where the agent is positioned within the safe zone and does not detect the boundary, the gradient is undefined. As we propose a decentralized multi-agent solution to this shepherding problem, the potential function is a way of representing locally observed information that will be communicated between agents.

## 4. Algorithm

### 4.1. Caging

We describe the algorithm from the perspective of an individual agent $a_{i}\in A$. Let $A_{n}$ and $H_{n}$, respectively, represent the neighbouring subsets of agents and herd members, which are located within the detection distance *d*_*d*_ from *a*_*i*_.

In the case where the agent is unable to detect any member of the herd, an arbitrary search method is applied (e.g. a random walk). We opt for an adaptation of the herd motion model presented in §3.1 to ensure neighbouring agents remain within communication range of each other. Agents communicate their observations of members of the herd to one another. Consequently, other agents who are unable to directly observe the herd, will become attracted to the neighbouring senders.

**Figure 3.**
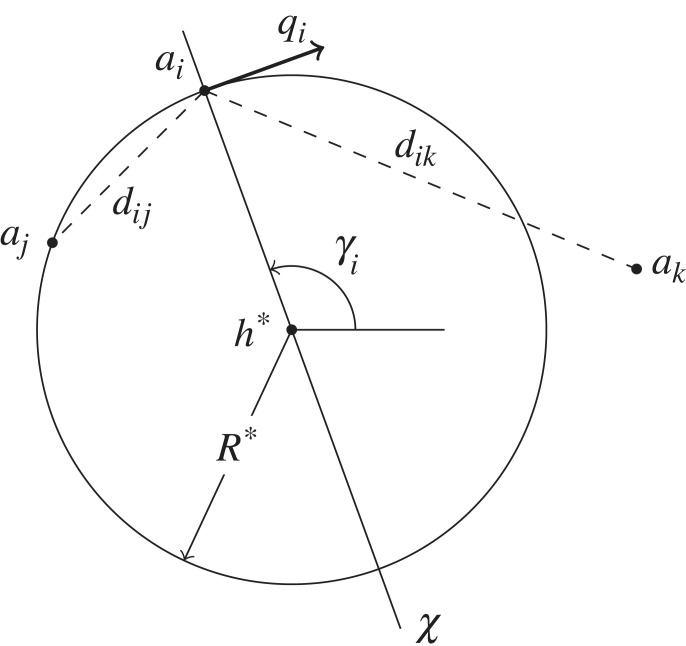
Illustration of the proposed caging method. Agent *a*_*i*_ follows tangential motion along the circle defined by the position of the closest herdable h* as the centre and R* as the radius. From the two closest neighbouring agents *a*_*j*_ and *a*_*k*_ of opposite sides of the axis *χ*, the agent *a*_*i*_ moves towards the neighbour which has the largest relative distance. Here, the motion vector ***q***_ *i*_ of *a*_*i*_ indicates clockwise rotation towards *a*_*k*_ as *d*_*ik*_ > *d*_*ij*_.

When members of the herd are directly detected, the agent attempts to reside at a given distance R* from the closest herdable $h^{*}\in H_{n}$. The agents should be close enough to observe, follow and quickly interact with the herd when needed. However, they should not be closer than *z*_*I*_, as this would otherwise induce unwanted stress on the herd. While approaching to and residing at the circular boundary of h*, the agent *a*_*i*_ attempts to remain equidistant from the two closest neighbouring agents *a*_*j*_ and *a*_*k*_ from opposite sides of the axis *χ* defined by the bearing *γ*_*i*_ ∈ [−*π*, *π*) of *a*_*i*_ from h*. As shown in figure 3, the neighbouring agents can be positioned anywhere in their respective half-plane.

**Figure 4.**
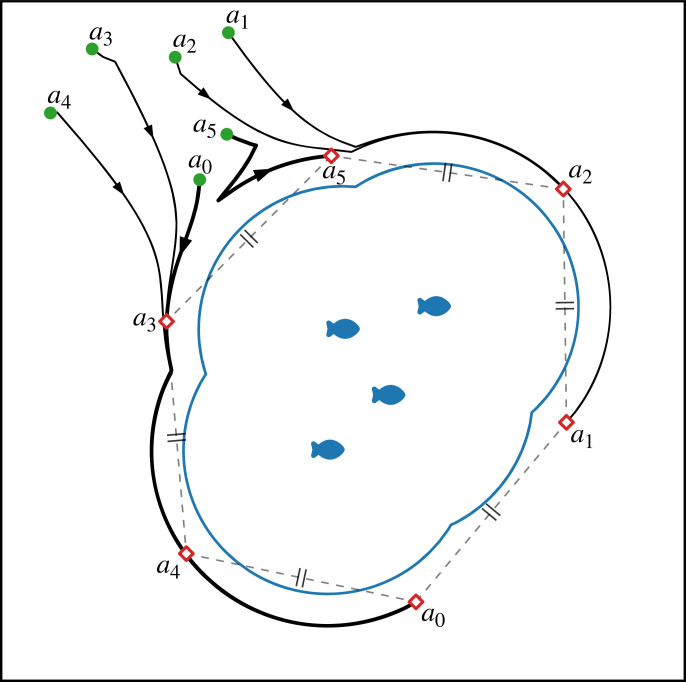
Trajectories (black arrows) of agents $A={\left\{a_{i}\right\}}_{i=0\ldots 5}$ caging a stationary herd of four (blue fish). The agents are initially located (green dots) in a box of density *ρ*_*a*_ near the herd, from which they establish and maintain an equidistant caged formation in steady state (red diamonds). At all times, the agents remain at a distance R* based on the herd’s repulsive zone (blue contour) of radius *z*_*I*_. The highlighted (thicker arrows) trajectories of *a*_0_ and *a*_5_, respectively, show how agents could either continuously move by the same direction or change directions due to the movement of neighbours.

Every agent attempts to maintain a distance of R* from the closest herd member, while moving to position themselves at equal distance from the two neighbouring agents. In other words, the agents move along the boundary of the union of circles defined by the positions of every herd member as the centre points and R* always as the radius. Figure 4 shows how the agents follow this boundary, which can be partially seen by the black trajectories (for illustrative purposes, the herd does not move). To provide full flexibility, an agent should be able to eventually re-encounter any point on the boundary after moving along the boundary in the same direction for enough time. This means that the union of circles is a connected set.

**Assumption 4.1.**

The union of the circles, where the centre points are the positions of every herd member h∈H and the radii are R*, is a connected set.

In order to approach and rotate along a circular path of radius R*, we apply the following method to compute the agent’s desired orientation $\;\hat{\;\theta }$ as proposed in [54]:

4.1 $$\;\hat{\;\theta }=\gamma_{i}+\vartheta \left(\frac{\pi }{2}+\arctan(\kappa (d^{*}-R^{*}))\right),$$

where ϑ∈{−1,1} determines the direction, i.e. clockwise (ϑ=−1) or counterclockwise (ϑ=1), *κ* > 0 influences the rate of transition between moving towards and moving along h*, and d* is the distance from *a*_*i*_ to h*.

To obtain the cage formation, the value of ϑ is computed based on the relative distances to the neighbouring agents. We define the subsets of neighbouring agents $A_{j}\subseteq A_{n}$ and $A_{k}\subseteq A_{n}$ divided by the axis *χ* as follows:

$$A_{j}=\{j|a_{j}\in A_{n}\wedge 0<\mathrm{sgn}(\gamma_{i})\cdot (\gamma_{i}-\gamma_{\;j})\leq \pi \}$$

and

$$A_{k}=A_{n}-A_{j}$$

with *γ*_*j*_ as the bearing of neighbouring agent *a*_*j*_ from h*. The shortest distances to each neighbour subset, $d_{ij}=g_{i}(A_{j})$ and $d_{ik}=g_{i}(A_{k})$, are computed by

$$g_{i}(U)=\left\{ \begin{array}{cc} \min_{u\in U}\|p_{u}(t)-p_{i}(t)\|\; & \mathrm{if}\;U\neq \emptyset \;\\ 2z_{I}\; & \mathrm{otherwise},\; \end{array}\right.\;$$

where the agent assumes a distance of 2*z*_*I*_ in the case where the neighbour subset is empty. The direction ϑ is then given by

$$\vartheta =\left\{ \begin{array}{cc} \mathrm{sgn}(\gamma_{i})\; & \mathrm{if}\;d_{ij}<d_{ik}\;\\ -\mathrm{sgn}(\gamma_{i})\; & \mathrm{otherwise}.\; \end{array}\right.$$

The orientation $\hat{\theta }$ of the motion vector to move along the circular path of h* is computed with equation (4.1) based on the direction ϑ. We define the magnitude of this motion vector as *η* = (1/2)|*d*_*ij*_ − *d*_*ik*_|, so that two agents moving towards each other will eventually reach a stable solution. This motion vector $\left\langle \hat{\theta };\eta \right\rangle$ is then added with the predicted motion vector of the closest member of the herd, such that the cage formation is at the appropriate relative distance of the herd. Each member of the herd is predicted to move at mean velocity ${\bar{v}}_{h}$ in its current orientation. Thus, the predicted motion vector of the nearest herd member is $\left\langle \theta_{h^{*}}(t);{\bar{v}}_{h^{*}}(t)\right\rangle$. Note that *η* = 0 when the agent has positioned itself at equal distance from both neighbours, which results in the agent aligning its orientation and velocity with the herd. However, when the agent is not equidistant to *a*_*j*_ and *a*_*k*_, we ensure that the maximum velocity is not exceeded as the magnitude *η* is upper bounded by $-{\bar{v}}_{h^{*}}(t)\cos(\theta_{h^{*}}(t)-\hat{\theta }(t))+\sqrt{{\bar{v}}_{h^{*}}^{2}(t)(\cos^{2}(\theta_{h^{*}}(t)-\hat{\theta }(t))-1)+v_{\max}^{2}}$, with *v*_max_ as the maximum linear velocity of the robotic agents (see appendix A for a derivation of the bound).

### 4.2. Shepherding

The idea behind the proposed algorithm is based on each individual agent adaptively changing between two caging formations, where one causes a repulsive force on the herd and the other does not.

Each agent *a*_*i*_ observes the gradient of the local potential $\nabla f\;(p_{i})$ as described in the problem formulation (see §3.3). A message containing a unique agent identifier, version and the local gradient is communicated to all nearby neighbours within the communication range *d*_*c*_. Whenever a new local gradient is observed, the agent updates its own version and sends a new message. The received messages are then filtered so that only messages with the newest version of each agent remain. All filtered messages are then forwarded to their nearby neighbours. The local gradients of the filtered messages are combined as $q_{f}=\sum_{\;j}(\nabla f\;(p_{j})/\left\|\nabla f\;(p_{j})\right\|)$, with *a*_*j*_ as an agent of which the gradient (with most recent version) is defined and received by *a*_*i*_. In case any *a*_*j*_ exist (including *a*_*i*_), we attempt to steer the herd in the direction of *q*_*f*_. In this step, we have assumed that the agents share a global coordinate system. This allows the agents to reach consensus in the mean direction when combining multiple locally observed potential gradients.

**Assumption 4.2.**

All agents are able to use a global coordinate system when communicating about orientations.

**Figure 5.**
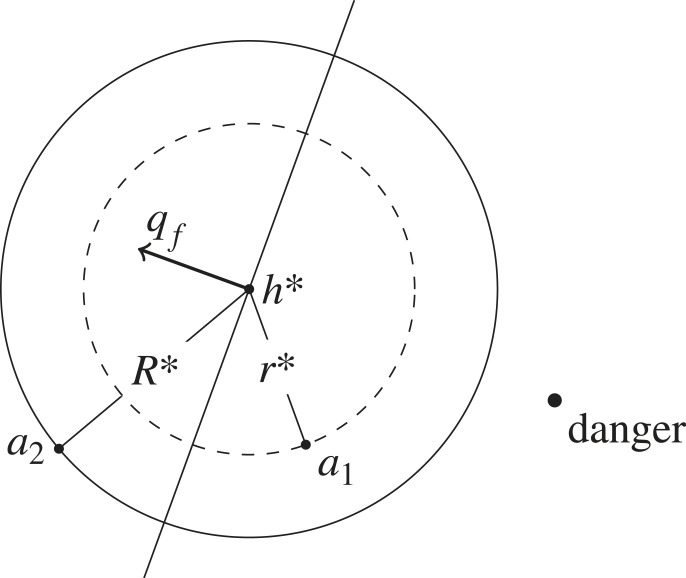
Illustration of the proposed shepherding method. Agent *a*_1_ is located behind the closest herdable h*, in perspective of the mean orientation *θ*_*f*_ (illustrated by the corresponding vector *q*_*f*_ with perpendicular axis). Therefore, *a*_1_ positions itself at a distance of r* from h*. Agent *a*_2_, positioned in front of h*, remains at a distance of R*.

Based on the aversive behaviour described in §3.1, a robotic agent is capable of triggering a herd individual to move in the direction opposite along the axis between the herd individual and this agent, when the relative distance is lower than *z*_*I*_. If an agent is positioned behind all nearby herd members, in the direction of *q*_*f*_, then it performs the caging algorithm to remain at the circular boundary of the closest herdable at a distance of r*, with r* < *z*_*I*_ < R*. The distance r* can be dynamically changed, as long as the upper bound is satisfied. In our experiments, we use a fixed value. In the other case, positioned in front of the nearby herd, the agent executes the caging algorithm with a distance of R*. Figure 5 shows how agents remain at different distances from the herd, based on their position relative to the direction of *q*_*f*_. More specifically, agent *a*_*i*_ steers by a caging with a radius of r* if the smallest angle $\gamma_{i}^{h}$ between the agent and each individual of the nearby herd $h\in H_{n}$ is greater than *π*/2:

$$\arctan(\sin(\theta_{f}-\gamma_{i}^{h}),\cos(\theta_{f}-\gamma_{i}^{h}))>\frac{\pi }{2},$$

with *θ*_*f*_ as the orientation of the vector *q*_*f*_.

If no messages of gradients are received, the agents remain in a caging formation at the distance R*.

## 5. Results

First, in §5.1, we study the behaviour of a simulated herd, when there are no robotic agents nearby, following the four different models of collective motion. We vary the most relevant parameters in regards to cohesiveness of the group [16], the widths *z*_*O*_ and *z*_*A*_ of the orientation and attraction zones, respectively, in order to examine in which parameter range assumption 4.1 holds. Afterwards in §5.2, we run an experiment with agents deployed in the environment that follow the proposed caging algorithm. We consider the same models of collective motion and varying parameter values (*z*_*O*_ and *z*_*A*_) of the herd, which allows us to verify if caging indeed only fails when assumption 4.1 does not hold. Finally in §5.3, we run experiments of the two aforementioned shepherding tasks: (i) agents steering the herd away from randomly appearing dangerous patches and (ii) agents keeping the herd inside a safe circular zone.

Results were obtained from simulation runs with 10 seeds. All experiments are run with the following parameters unless explicitly stated otherwise: *N*_*H*_ = 100, *z*_*I*_ = 19, *w*_*h*_ = *w*_*a*_ = *π*/2, $v_{\max}^{a}=4$, $v_{\max}^{h}=2$, *d*_*d*_ = *d*_*c*_ = 3 *z*_*I*_, *σ*_*a*_ = *σ*_*h*_ = 0.05, *α*_*R*_ = 100, *α*_*O*_ = 50, *α*_*A*_ = 1 and *α*_*I*_ ∈ [500, 2500].

### 5.1. Collective motion models

The herd is simulated, in the absence of any robotic agents, under the different models of collective motion: (*a*) metric, (*b*) topological, (*c*) visual and (*d*) long-range. We simulate the herd in two-dimensional open space environment. To facilitate immediate interaction between the *N*_*H*_ individuals at the beginning of a simulation, they are placed within a square region of a size (*N*_*H*_/*ρ*_*h*_)^1/2^ with initial density *ρ*_*h*_ = 0.01. Each individual’s position and moving direction are initially uniformly distributed.

We aim to study which parameter values lead to fragmentation of the herd, since this would prohibit agents from successfully caging the herd. Thus, we say that the herd is fragmented when assumption 4.1 does not hold. As previously described, a graph can be constructed where the vertices are the positions of every herd member, and there is only an edge between two vertices if the relative distance is lower than or equal to R*. Based on this graph, the degree of fragmentation is measured as the number of groups *N*_*G*_ where each distinct group is connected. We vary the respective widths *z*_*O*_ and *z*_*A*_ of the orientation and attraction zones over [0, 100]. For the parametric models (topological and long-range), we chose values that produce most similar results of fragmentation to the other two models. This allows us to make a fair comparison between models. We set *k* = 50 in the topological model, since lower values of *k* lead to only higher probabilities of fragmentation, as shown in [16]. We verify this by running simulations for *k* ∈ {10, 25, 50} (see appendix C). This led them to proposing the long-range model, for which we use their proposed values of *k* = 7 and *λ* = 0.1. As visual reconstruction, a ray-casting algorithm is used in this paper where the individuals are simulated guppy fish based on physical measurements.

In addition to the number of groups, we examine the relative area coverage *A*_*r*_ which is computed as the ratio of the areas of the convex hulls of the herd at convergence time *T* over initial time *t*_0_. Related to this, the minimum number of robots needed to form a caging pattern *N*_*A*,min_ is computationally estimated by placing agents on the boundary of the union of circles, described in assumption 4.1, at a maximum distance of 2*z*_*I*_ from one another. Based on these results, we can test our hypothesis that the proposed caging algorithm fails if and only if assumption 4.1 does not hold or there was an insufficient number of robotic agents deployed.

**Figure 6.**
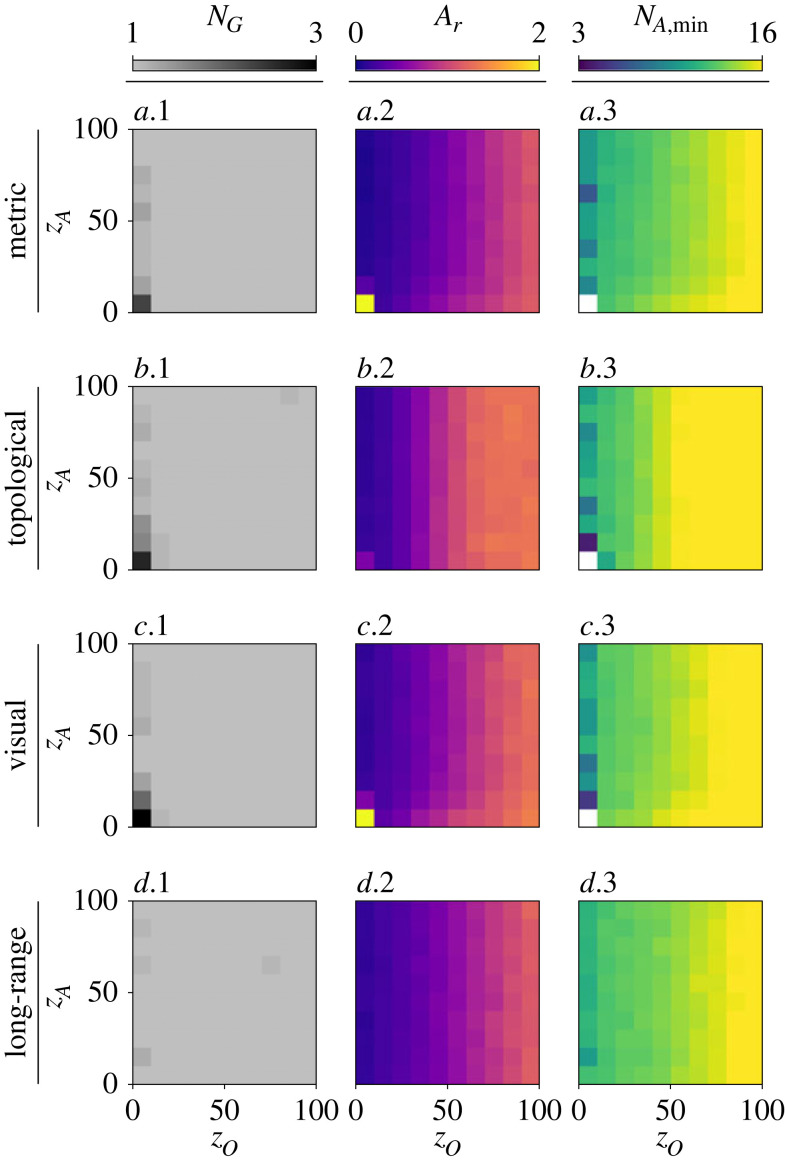
Quantitative herd measurements (*N*_*H*_ = 100) obtained in simulation without robotic agents, following different models of collective motion: (*a*.1–3) metric, (*b*.1–3) topological with *k* = 50, (*c*.1–3) visual and (*d*.1–3) long-range with *k* = 7, *λ* = 0.1. For each model, the widths of orientation (*z*_*O*_) and attraction (*z*_*A*_) zones are varied. The number of groups *N*_*G*_ (*a–d*.1) shows that group fragmentation only occurs with for smaller values of *z*_*O*_, independent of the width of zone of attraction *z*_*A*_, for all models (although, the long-range model causes significantly less fragmentation). The relative area coverage *A*_*r*_ (*a–d*.2) increases with *z*_*O*_, mostly independent of *z*_*A*_. When the herd remains as one cohesive group, the theoretical minimum number of agents *N*_*A*,min_ needed to form a caging pattern (*a–d*.3) increases with *z*_*O*_ in correlation with the relative coverage *A*_*r*_ for each model respectively, since more agents are needed to cage a larger area. On the other hand, *N*_*A*,min_ is high when fragmentation occurs.

Figure 6 shows the aforementioned quantitative measurements for the four considered models, and varying the widths *z*_*O*_ and *z*_*A*_ of the orientation and attraction zones, respectively. Figure 6*a–c.1* shows that the herd becomes fragmented (*N*_*G*_ > 1) for lower values of the orientation width (*z*_*O*_ < 10), using a metric, topological, or visual model. In this range of *z*_*O*_, the herd fragments into the most groups when *z*_*A*_ is lowest. As opposed to the other models, figure 6*d.1* shows that fragmentation is unlikely to occur with the long-range model, independent of *z*_*O*_ and *z*_*A*_. Furthermore, we see in figure 6*a–d.2* that the relative area *A*_*r*_ is also dependent on the width of the orientation zone *z*_*O*_, while mostly independent of *z*_*A*_. The herd becomes denser than its initial distribution (*A*_*r*_ < 1) for the lowest values of *z*_*O*_. Increasing *z*_*O*_ leads to increasing the relative area coverage until *A*_*r*_ is approximately equal to 1 or slightly higher. Note how in the topological model *A*_*r*_ eventually stabilizes once *z*_*O*_ is approximately higher than 60. Evidently, when the orientation zone is so large that each individual aligns its orientation with all its neighbours, the herd will maintain its initial area. On the contrary, when *z*_*O*_ is small enough so that neighbours also appear in the attraction zone, the herd will become more compact. As the relative area *A*_*r*_ increases with *z*_*O*_, so does *N*_*A*,min_ (figure 6*a–d.3*) as more robotic agents are needed to form a caging pattern where consecutive robots should remain at a distance lower than 2*z*_*I*_. Note that the theoretical minimum of number of agents is not computed when the herd is fragmented (*N*_*G*_ > 1). In the figure, a white square means no possible value as all seeds contain fragmentation.

### 5.2. Caging

**Figure 7.**
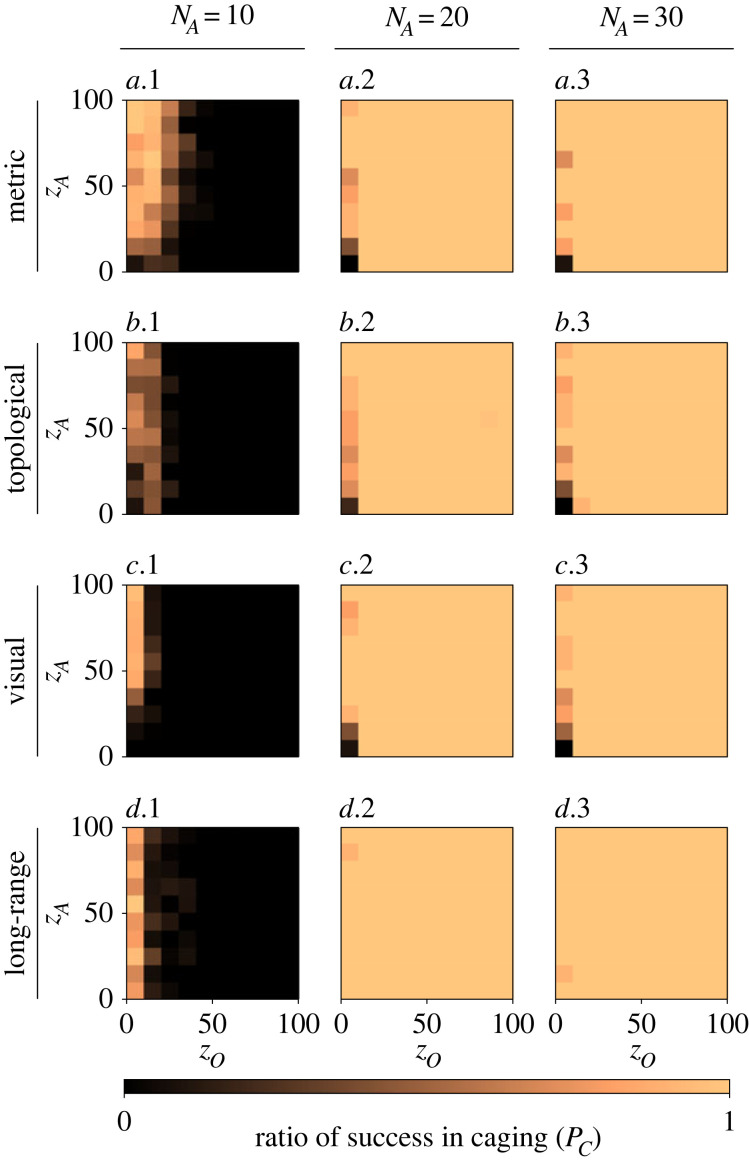
The ratio of robots successfully caging a herd of size *N*_*H*_ = 100, following different models of collective motion: (*a*.1–3) metric, (*b*.1–3) topological with *k* = 50, (*c*.1–3) visual and (*d*.1–3) long-range with *k* = 7, *λ* = 0.1. Additionally, different sizes of the robot swarm *N*_*A*_ are studied: (*a–d*.1) *N*_*A*_ = 10, (*a–d*.2) *N*_*A*_ = 20 and (*a–d*.3) *N*_*A*_ = 30. For each combination of motion model and swarm size, the widths of orientation (*z*_*O*_ ) and attraction (*z*_*A*_) zones are varied. Although the herd remains cohesive in the range of *z*_*O*_ > 10, caging with *N*_*A*_ = 10 robots is entirely unsuccessful in this range since more agents are required (*N*_*A*_ = 10 < *N*_*A*,min_). On the other hand, deploying *N*_*A*_ = 20 > *N*_*A*,min_ robots guarantees successful caging in every seed. The same results are found for *N*_*A*_ = 30, which shows that adding redundant robots has no negative effects on the collective performance. In the range of *z*_*O*_ ≤ 10, the theoretical lower bound *N*_*A*,min_ is satisfied for all considered *N*_*A*_, but fragmentation of the herd may occur, and thus caging is not always successful (except for the long-range model where significantly less fragmentation occurs).

In this second experiment, we placed *N*_*A*_ robotic agents following the proposed caging algorithm in the same environment as the herd. The swarm of *N*_*A*_ agents is placed in a square region based on initial density *ρ*_*a*_ = 0.01, at a random but detectable position from the school (i.e. within distance *d*_*d*_). Figure 7 shows the ratio of successfully caging a herd with different number of agents (i) *N*_*A*_ = 10, (ii) *N*_*A*_ = 20 and (iii) *N*_*A*_ = 30, and for the different collective motion models of the herd.

Figure 7*a–d.*1 shows that, in the range of *z*_*O*_ > 10, deploying *N*_*A*_ = 10 robotic agents is inadequate to successfully cage a herd of size *N*_*H*_ = 100. This was expected as *N*_*A*_ = 10 < *N*_*A*,min_ (see the first column inside the colour-grids of figure 6*a–d.*3). However, when a sufficient number of robots is deployed (*N*_*A*_ = 20 > *N*_*A*,min_), caging is successful in every seed with *z*_*O*_ > 10 (figure 7*a–d.*2). Figure 7*a–d.*3 shows that adding a redundant number of robots to the task does not decrease the ratio of success. This is an important quality of the algorithm, as the theoretical minimum number of agents to be deployed is usually unknown *a priori*. For any *N*_*A*_ ∈ {20, 30}, the theoretical lower bound *N*_*A*,min_ is satisfied when fragmentation of the herd does not occur in the range of *z*_*O*_ ≤ 10. However, the herd is not guaranteed to remain as one cohesive group in this range of *z*_*O*_, in which case the robots will fail to cage properly. In this experiment, the agents consistently try to remain at a distance of R* > *z*_*I*_ from the herd, which means that caging does not prohibit the herd from fragmenting. Small adaptations to the proposed caging algorithm could prevent fragmentation; for instance the agents should maintain the maximum relative distance of 2*z*_*I*_ between agents instead of following the herd’s movement. As an exception, the robot swarm is still successful in this range (figure 7*d.*2–3) as fragmentation of the herd is unlikely to occur (figure 6*d.*1). In all cases, we find that failure in caging the entire herd occurs if and only if the herd is fragmented, or there is an inadequate number of robots deployed based on the theoretical minimum.

**Figure 8.**
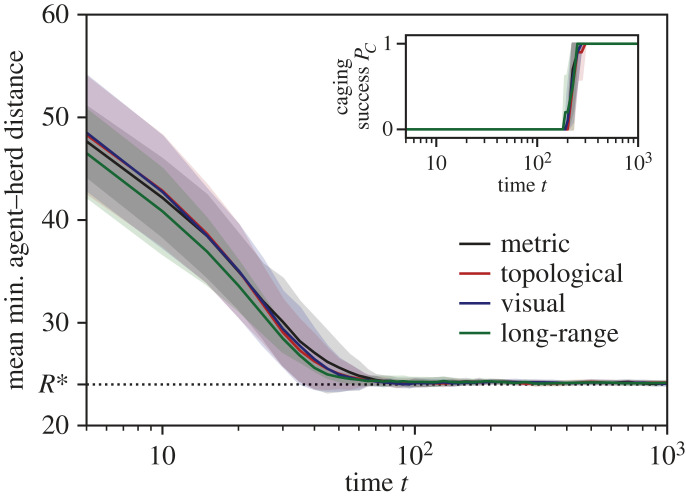
The average minimum distance between the robotic agents and the herd, and ratio of success in caging *P*_*C*_ in the inset, over time *t* from 0 to 10^3^ in log scale with *N*_*H*_ = 100, *N*_*A*_ = 20, *z*_*O*_ = 50 and *z*_*A*_ = 50. The mean is drawn as a solid line and the standard deviation as shaded area. The desired convergence value R* of the minimum agent–herd distance is indicated on the *y*-axis and drawn as a dashed horizontal line. For each plot, measurements of four different models are shown: (black) metric, (red) topological, (blue) visual and (green) long-range.

In order to observe the time needed for the robot swarm to converge to a stationary caging formation, we measure the average of minimum relative distances between each robotic agent and their respective closest individual of the herd ‖*p*_*a*_(*t*) − *p*_h*_(*t*)‖. As described in §4.1, the agents should approach a distance of R*, which is lower bounded by *z*_*I*_ to account for prediction errors of the herd’s movement. Figure 8 shows that the robots are able to converge to R* over time for every collective motion model of the herd. More specifically, the average minimum distance between agents and herd reaches R* at approximately *t* = 10^2^. After this time, the robots spread out around the herd and eventually reach a successful caging formation (*P*_*C*_ = 1).

### 5.3. Shepherding

To examine the proposed shepherding algorithm, we simulate two different tasks: (i) dangerous patches and (ii) safe enclosure as described in §3.3. We study the performance of our shepherding algorithm for the four models of collective motion that have been presented in previous sections: metric, topological, visual and long-range. Results in the paper are shown for a herd size *N*_*H*_ = 100.

#### 5.3.1. Dangerous patches

**Figure 9.**
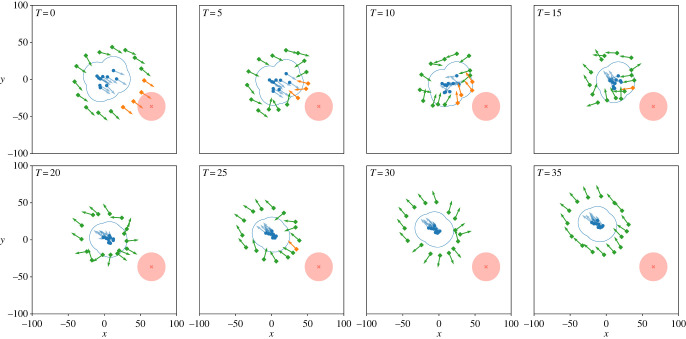
Visualization of robotic agents (green or orange diamonds) shepherding the herd (blue dots) away from a dangerous patch (red circle). When a robot has detected a danger, it is coloured orange and green otherwise. The orientation of each individual is represented by an arrow. The blue contour is the union of each herd member’s circular zone with radius *z*_*I*_. In other words, only agents that are positioned in the blue contour generate a repulsive force onto the herd. At *t* = 0, agents detect the danger. Next (*t* = 5 to 20), the agents positioned between the herd and the danger now execute the caging algorithm with distance r* < *z*_*I*_, while the other agents remain at R* > *z*_*I*_. This causes the herd to re-orientate and move away from the danger. Once the danger is enough far away, all agents occupy a distance of R* from the herd.

Figure 9 visualizes the first scenario over time, where a dangerous patch is placed in front of the herd. At *t* = 0, the robots are in a caging formation, remaining at a distance R* > *z*_*I*_ from the herd. At this time, some robots detect the danger and locally communicate this to their neighbours. The appropriate subset of robots now apply a cage formation at a distance r* < *z*_*I*_, in order to push the herd in the opposite direction of the danger. In the following time steps, the herd changes movement and other robots also redirect their orientation. The herd and robots move away together from the danger. Once the danger is far enough away (the danger is out of the detection distance *d*_*d*_ at *t* = 20), each robot transitions back to caging at the distance R*, which is fully established again at *t* = 35.

**Figure 10.**
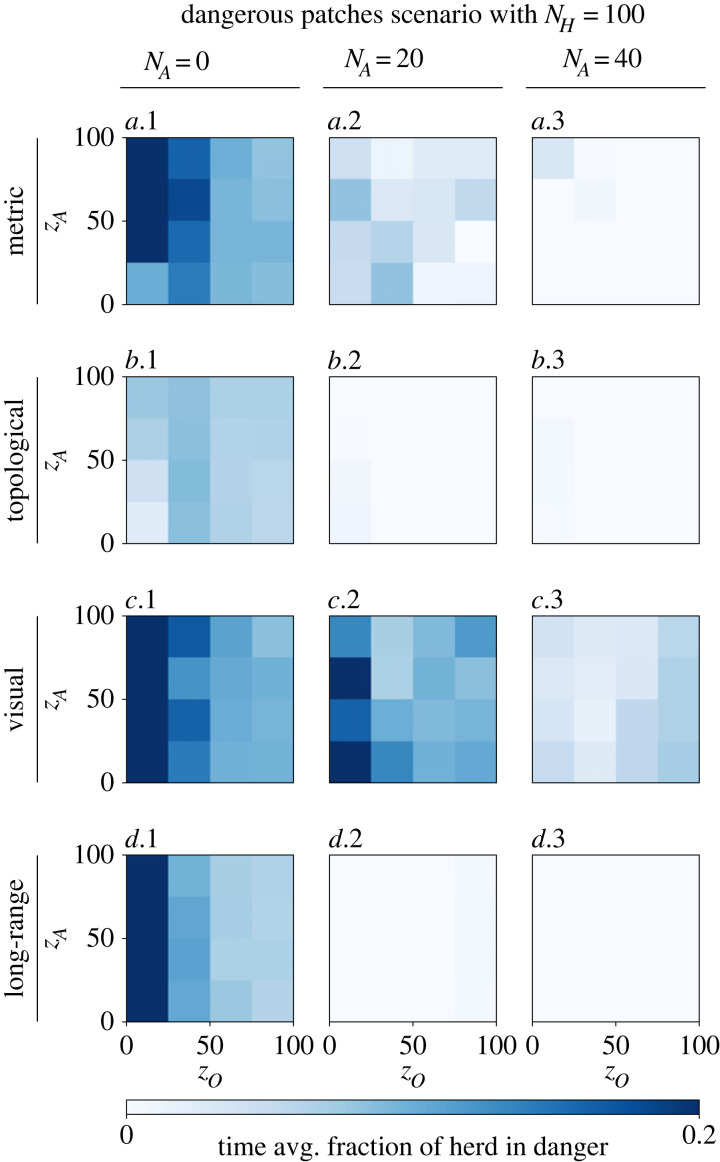
Fraction of the herd (*N*_*H*_ = 100) located in dangerous patches, averaged over 2000 time steps, and following different models of collective motion: (*a*.1–3) metric, (*b*.1–3) topological with *k* = 50, (*c*.1–3) visual and (*d*.1–3) long-range with *k* = 7, *λ* = 0.1. The left column (*a–d*.1) *N*_*A*_ = 0 shows the results where no robots are present and thus no shepherding takes place. In the other columns, a shepherding robot swarm of sizes (*a–d*.2) *N*_*A*_ = 20 and (*a–d*.3) *N*_*A*_ = 40 are studied. For each combination of motion model and swarm size, the widths of orientation (*z*_*O*_) and attraction (*z*_*A*_) zones are varied. Without shepherding, a certain fraction (non-zero) of the herd is on average in danger for every model and values of *z*_*O*_ and *z*_*A*_. The topological model results in the lowest endangered fraction, followed by the long-range model, while the metric and visual models both perform the worst. When modelled as topological or long-range, deploying 20 shepherding robots successfully ensures the safety of the entire herd. Adding more robots (*N*_*A*_ = 40) shows to only potentially improve performance: the metric-modelled herd is successfully shepherded for most values of *z*_*O*_ and *z*_*A*_; however, the visual-modelled herd remains endangered (although with low probability).

In order to observe whether the robotic agents are capable of repeatedly shepherding the herd in different directions, dangerous patches are placed in front of the herd after a certain time interval. Each time interval is newly sampled from a Gaussian distribution with mean 150 and standard deviation 25. An individual of the herd only detects the danger once it is located inside the patch, which is similar to animals encountering oil leaks and plastic pollution. When the herd is dense, multiple individuals may detect the danger at the same time. In some way this may be necessary, as the mean direction of the herd is hardly changed by the action of a single individual. Figure 10 shows the fraction of the herd that is located outside of the safe enclosure, on average over time. Without shepherding, the herd is endangered for any model of its collective motion. With 20 shepherding robots, the herd stays out of any danger when modelled as topological or long-range for any values of *z*_*O*_ and *z*_*A*_. For the metric model, the mean fraction is significantly decreased by deploying robots and eventually becomes zero with a robot swarm of size *N*_*A*_ = 40 for most values of *z*_*O*_ and *z*_*A*_. Although the performance of shepherding a herd that follows a visual model improves by adding more robots (*N*_*A*_ = 20 to 40), a certain fraction of the herd remains endangered. We argue that the models likely differ in speed of information diffusion, which influences the number of individuals that continues to move towards the danger. For the visual model, deploying additional robots may not improve information diffusion when those additional robots are positioned in an area occluded by others. We find that this issue only arises for larger herd sizes, as appendix D shows that for any collective motion model, a herd of size *N*_*H*_ = 10 remains safe when shepherded by robots following the proposed algorithm.

**Figure 11.**
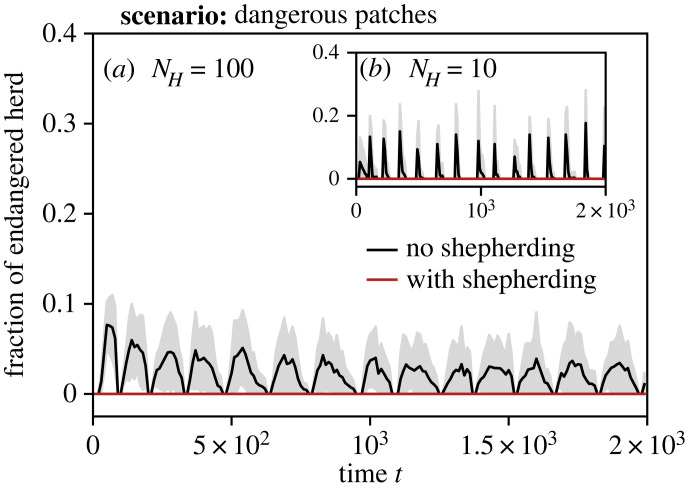
Fraction of herd located in dangerous patches over time *t*, deploying shepherding robots (red) or no robots (black). Results for sizes of the herd and robot swarm, respectively: (*a*) *N*_*H*_ = 100 with *N*_*A*_ = 40, (*b*) *N*_*H*_ = 10 with *N*_*A*_ = 20. The herd follows a metric model with *z*_*O*_ = 50 and *z*_*A*_ = 50. The mean is drawn as a solid line and the standard deviation as shaded area.

Figure 11 shows the fraction of the herd that is positioned in a dangerous patch at time *t*. We see that for both *N*_*H*_ = 100 and *N*_*H*_ = 10, a fraction of the herd is endangered without the aid of shepherding robots, while the herd remains safe at all times in the presence of robots following our proposed algorithm.

#### 5.3.2. Safe enclosure

**Figure 12.**
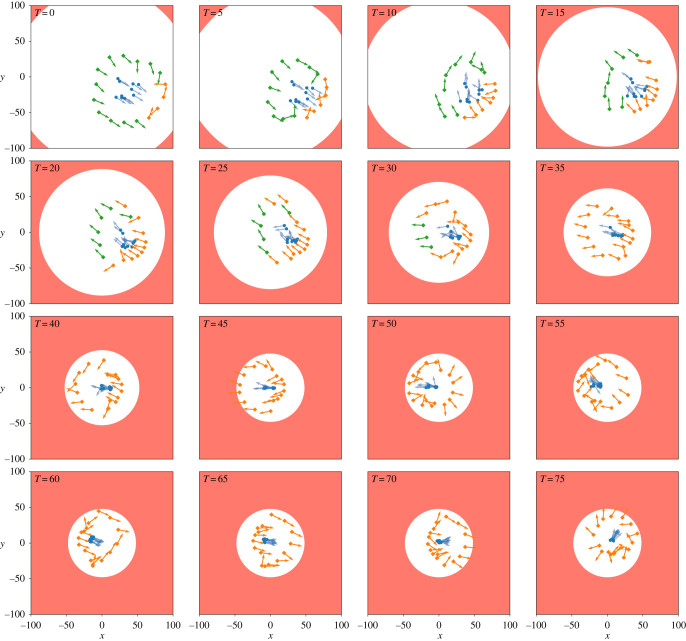
Visualization of robotic agents (green or orange diamonds) shepherding the herd (blue dots) to remain in the safe enclosure (white area). When a robot has detected a danger, it is coloured orange and green otherwise. The orientation of each individual is represented by an arrow. The blue contour is the union of each herd member’s circular zone with radius *z*_*I*_. In other words, only agents that are positioned in the blue contour generate a repulsive force onto the herd. Agents adapt and repeatedly switch in caging with a distance of r* < *z*_*I*_ or R* > *z*_*I*_, depending on how close the herd is to the boundary of the safe enclosure.

Figure 12 visualizes the second scenario over time, where the safe zone (white) shrinks until a certain area. At *t* = 0, the robots are in a caging formation, remaining at a distance R* from the herd. Some robots have detected the danger zone (red) for the first time and begin re-orientating. At *t* = 5, the robots push the herd away from the boundary of the safe zone in a certain direction, until the boundary is reached again (*t* = 50). At this time, the agents redirect the herd towards another direction. This pattern continues as the robots attempt to keep the herd in the centre of the safe zone.

**Figure 13.**
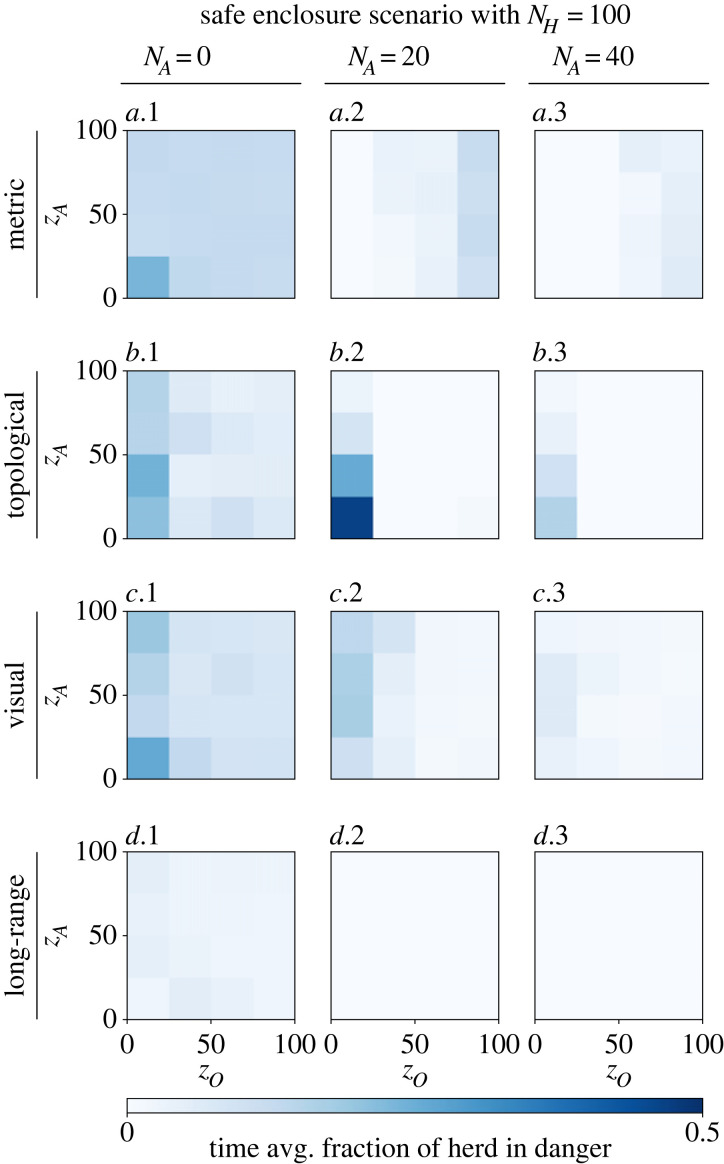
Fraction of the herd (*N*_*H*_ = 100) located outside of the safe enclosure, averaged over 2000 time steps, and following different models of collective motion: (*a*.1–3) metric, (*b*.1–3) topological with *k* = 50, (*c*.1–3) visual and (*d*.1–3) long-range with *k* = 7, *λ* = 0.1. The left column (*a–d*.1) *N*_*A*_ = 0 shows the results where no robots are present and thus no shepherding takes place. In the other columns, a shepherding robot swarm of sizes (*a–d*.2) *N*_*A*_ = 20 and (*a–d*.3) *N*_*A*_ = 40 are studied. For each combination of motion model and swarm size, the widths of orientation (*z*_*O*_) and attraction (*z*_*A*_) zones are varied.

The fraction of the herd that is located outside of the safe enclosure, on average over time, is presented in figure 13. Without shepherding, the herd is endangered when modelled as metric, topological and visual. Following the long-range model, however, only a small fraction of the herd is in danger. With 20 shepherding robots, the herd stays out of any danger when modelled as topological or visual for any *z*_*O*_ > 25. The long-range herd is continuously shepherded in the safe enclosure, with both 20 and 40 robots, for any values of *z*_*O*_ and *z*_*A*_. The metric model, however, performs the worst, as a fraction of the herd moves out of the safe enclosure when *z*_*O*_ is large.

**Figure 14.**
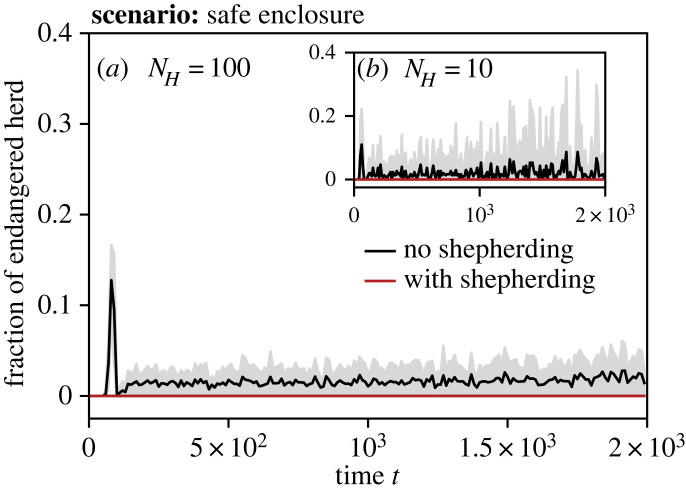
Fraction of herd located outside of the safe enclosure over time *t*, deploying shepherding robots (red) or no robots (black). Results for sizes of the herd and robot swarm, respectively: (*a*) *N*_*H*_ = 100 with *N*_*A*_ = 40, (*b*) *N*_*H*_ = 10 with *N*_*A*_ = 20. The herd follows a metric model with *z*_*O*_ = 50 and *z*_*A*_ = 50. The mean is drawn as a solid line and the standard deviation as shaded area.

Figure 14 shows the fraction of the herd that is positioned out of the safe enclosure at time *t*. We see again that for both *N*_*H*_ = 100 and *N*_*H*_ = 10, a fraction of the herd is endangered when no robots are present, while the herd remains safe at all times with the aid of shepherding robots. Most notably, there is a peak in the fraction of endangered herdables at the beginning of the scenario. This is most likely due to the herd being slower in detecting and reacting to the rapid shrinking of the safe enclosure.

## 6. Conclusion

In this paper, we proposed an algorithm on the individual level, which enables a swarm of robotic agents to shepherd an autonomous herd (i.e. a group following collective motion) away from dangers before the latter succumbs to these dangers. The robots rely on caging the herd at all times, so that any danger surrounding the herd can be immediately detected. We assume that herd individuals move away from robots that are closer than a certain distance. Based on this assumption, robots are capable of steering the herd in a desired direction by placing themselves close enough to the herd in the appropriate relative position. We studied the performance of the proposed caging algorithm in simulation for four different models of collective motion to simulate the herd: (i) metric, (ii) topological, (iii) visual and (iv) long-range. We found that when the herd remains cohesive (i.e. information can be passed between every pair of individuals in the herd), the robots are always capable of caging successfully if an appropriate number of robots is deployed. The required number of robots can be computed from Monte Carlo simulations if the collective motion model of the herd is known. Otherwise, it is best to overestimate the number as the performance of the algorithm does not decrease by deploying redundant robots. For parameters where the herd remains cohesive following a metric model, we examined the performance of robots shepherding by caging. We defined two different shepherding task scenarios: a first where dangerous patches appear with a probability over time, and a second where the herd should remain in a safe circular area. Simulation results for herds of sizes 10 and 100 both show that shepherding prevents any individual of the herd from encountering a danger. Without shepherding, a fraction of the herd is endangered. To the best of our knowledge, equipping robots with the proposed algorithm results in the first decentralized multi-robot system only using local observations and communication capable of shepherding a herd by caging. Future work includes simulating the motion of the herd by trained models of real animal trajectories, and eventually real-life experiments.

## Figures and Tables

**Figure 15.**
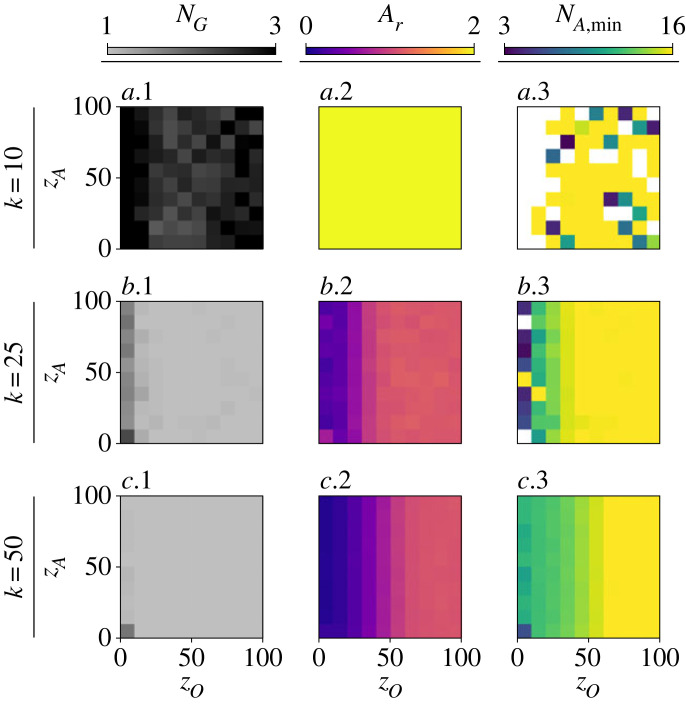
Quantitative herd measurements (*N*_*H*_ = 100) obtained in simulation without robotic agents, following the topological model with different values of *k* nearest neighbours: (*a*.1–3) 10, (*b*.1–3) 25, (*c*.1–3) 50. For each model configuration, the widths of orientation (*z*_*O*_) and attraction (*z*_*A*_) zones are varied. The left column (*a*–c.1) shows the number of groups *N*_*G*_, the middle column (*a–c*.2) shows the relative area coverage *A*_*r*_, and the right column (*a–c*.3) shows the theoretical minimum number of agents *N*_*A*,min_ needed to form a caging pattern.

**Figure 16.**
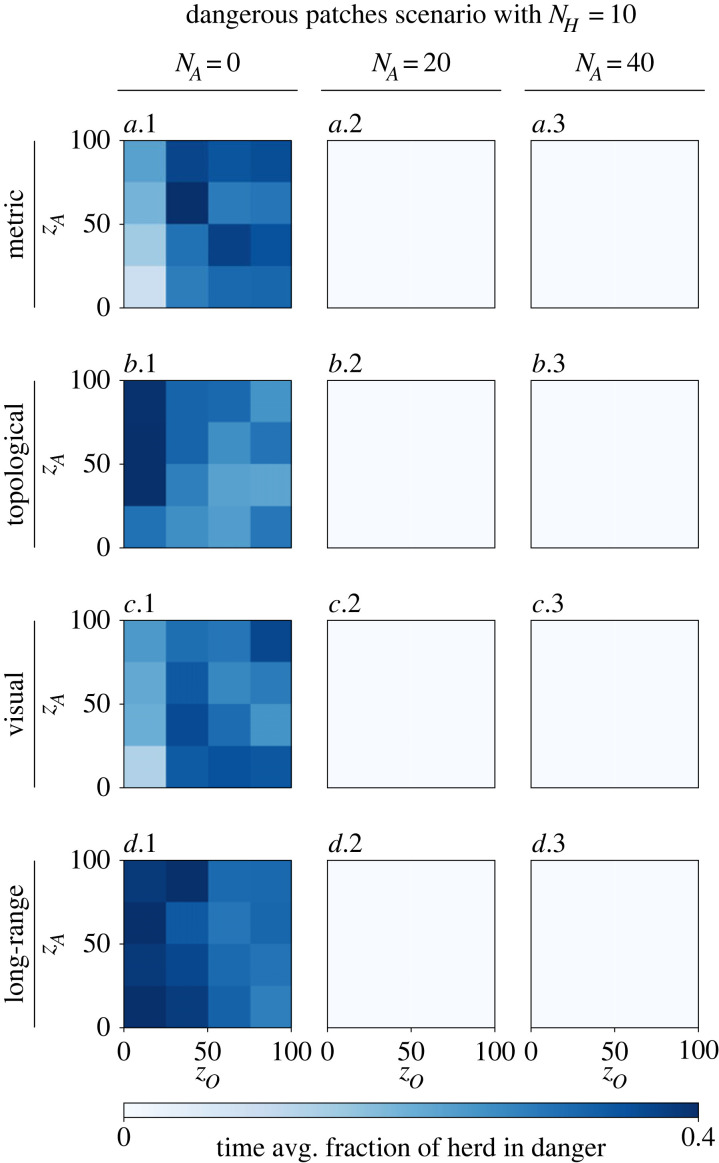
Fraction of the herd (*N*_*H*_ = 10) located in dangerous patches, averaged over 2000 time steps, and following different models of collective motion: (*a*.1–3) metric, (*b*.1–3) topological with *k* = 50, (*c.*1–3) visual and (*d*.1–3) long-range with *k* = 7, *λ* = 0.1. The left column (*a–d*.1) *N*_*A*_ = 0 shows the results where no robots are present and thus no shepherding takes place. In the other columns, a shepherding robot swarm of sizes (*a–d*.2) *N*_*A*_ = 20 and (*a–d*.3) *N*_*A*_ = 40 are studied. For each combination of motion model and swarm size, the widths of orientation (*z*_*O*_) and attraction (*z*_*A*_) zones are varied.

## Data Availability

Zip file named ‘submission-code-rsi-nodata.zip’ contains Python code to reproduce the experiments and data [55].

## Appendix A. Bound on the motion vector in caging to not exceed the maximum linear velocity

The motion vector *q* of an agent following the proposed caging algorithm described in §4.1 is the summation of vectors $\left\langle \hat{\theta };\eta \right\rangle$ and $\left\langle \theta_{h^{*}};{\bar{v}}_{h^{*}}\right\rangle$. We now define an upper bound on *η* such that ‖*q*‖ ≤ *v*_max_. This allows for the robot to move at maximum velocity, while maximizing its contribution to caging (i.e. by setting *η* to its upper bound). The value of *η* indicates how much velocity can be used on establishing a caging pattern, while replicating the movement of the nearest herd member *h**. Hence, it is required that the maximum linear velocity of the robot *v*_max_ is higher than the herd’s.

Let $q_{h,x}={\bar{v}}_{h^{*}}\cos(\theta_{h^{*}})$ and $q_{h,y}={\bar{v}}_{h^{*}}\sin(\theta_{h^{*}})$. It follows that

$$q=\langle q_{h,x}+\eta \cos(\;\hat{\;\theta });q_{h,y}+\eta \sin(\;\hat{\;\theta })\rangle .$$

Thus, a bound on the velocity is defined as

$$\begin{array}{rl} & v_{\max}^{2}\geq {\left(q_{h,x}+\eta \cos(\;\hat{\;\theta })\right)}^{2}+{\left(q_{h,y}+\eta \sin(\;\hat{\;\theta })\right)}^{2},\\ & v_{\max}^{2}\geq \eta^{2}+2\eta (q_{h,x}\cos(\;\hat{\;\theta })+q_{h,y}\sin(\;\hat{\;\theta }))+q_{h,x}^{2}+q_{h,y}^{2}\;\;\;\;\;\;\;\;\;\;\;\;\;\;\;\;\;\;\;\\ \mathrm{and}\;\;\;\;\;\;\;\; & 0\geq \eta^{2}+2\eta (q_{h,x}\cos(\;\hat{\;\theta })+q_{h,y}\sin(\;\hat{\;\theta }))+({\bar{v}}_{h^{*}}^{2}-v_{\max}^{2}). \end{array}$$

We can rewrite $q_{h,x}\cos(\hat{\theta })+q_{h,y}\sin(\hat{\theta })$ as follows:

$$\begin{array}{rl} & ={\bar{v}}_{h^{*}}\cos(\theta_{h^{*}})\cos(\;\hat{\;\theta })+{\bar{v}}_{h^{*}}\sin(\theta_{h^{*}})\sin(\;\hat{\;\theta })\\ & ={\bar{v}}_{h^{*}}\cos(\theta_{h^{*}}-\;\hat{\;\theta }). \end{array}$$

Substituting this in the upper bound:

$$\begin{array}{rl} & 0\geq \eta^{2}+2\eta {\bar{v}}_{h^{*}}\cos(\theta_{h^{*}}-\;\hat{\;\theta })+({\bar{v}}_{h^{*}}^{2}-v_{\max}^{2}),\\ & \eta \leq -{\bar{v}}_{h^{*}}\cos(\theta_{h^{*}}-\;\hat{\;\theta })\pm \sqrt{{\bar{v}}_{h^{*}}^{2}\cos^{2}(\theta_{h^{*}}-\;\hat{\;\theta })-({\bar{v}}_{h^{*}}^{2}-v_{\max}^{2})}\\ \mathrm{and}\;\;\;\;\; & \eta \leq -{\bar{v}}_{h^{*}}\cos(\theta_{h^{*}}-\;\hat{\;\theta })\pm \sqrt{{\bar{v}}_{h^{*}}^{2}(\cos^{2}(\theta_{h^{*}}-\;\hat{\;\theta })-1)+v_{\max}^{2}}. \end{array}$$

After root analysis, to ensure that *η* is positive, we find that

$$\eta \leq -{\bar{v}}_{h^{*}}\cos(\theta_{h^{*}}-\;\hat{\;\theta })+\sqrt{{\bar{v}}_{h^{*}}^{2}(\cos^{2}(\theta_{h^{*}}-\;\hat{\;\theta })-1)+v_{\max}^{2}}.$$

## Appendix B. Simulation parameters

Table 1 summarizes all parameters used in the simulation experiments, where topological and long-range are, respectively, abbreviated as top and l–r.

**Table 1.** Simulation parameters.

symbol	parameter	value	association
*w*_*h*,max_/*w*_*a*,max_	max. angular velocity	$\frac{\pi }{3}/\frac{\pi }{2}$ $\text{rad}\;{\text{s}}^{-1}$	herd/robot
*v*_*h*,max_/*v*_*a*,max_	max. linear velocity	$2/4\;\text{cm}\;{\text{s}}^{-1}$	herd/robot
*σ*_*h*_/*σ*_*a*_	Gaussian orientation noise	0.05/0.05 rad	herd/robot
*z*_*R*_	radius of repulsion zone	1 cm	herd
*z*_*O*_	radius of orientation zone	[10,100] cm	herd
*z*_*A*_	radius of attraction zone	[10,100] cm	herd
*k*	nearest neighbours	50 (top), 7 (l–r)	herd
*λ*	average of Poisson distribution (l–r)	0.1	herd
*z*_*I*_	radius of aversive zone	19 cm	herd
*α*_*R*_	weight of repulsion interaction	100	herd
*α*_*O*_	weight of repulsion interaction	50	herd
*α*_*A*_	weight of repulsion interaction	1	herd
*α*_*I*_	weight of repulsion interaction	[500, 2500]	herd
*d*_*d*_	robot-detection radius	57 cm	robot (cage)
*R**	distance robot–herd	24 cm	robot (cage)
*κ*	transition rate	0.2	robot (cage)
*r**	distance robot–herd	17 cm	robot (shepherd)
*d*_*c*_	communication radius	57 cm	robot (shepherd)
*d*_*z*_	danger-detection radius	40 cm	robot (shepherd)
*N*_*H*_	number of herd members	[10, 100]	herd
*N*_*A*_	number of robotic agents	[0, 10, 20, 30, 40]	robot

## Appendix C. Quantitative herd measurements of the topological model for different values of *k* nearest neighbours

Figure 15 shows that a topological model with *k* = 10 leads to group fragmentation for any widths of the zones. When *k* is set to 25, the herd remains cohesive for most values of *z*_*O*_ and *z*_*A*_. Finally, for *k* = 50, the topological model produces results most similar to those of the other three models (figure 6).

## Appendix D. Shepherding a herd of 10 individuals

In figure 16, the average fraction of endangered herd over time is shown for all four herd models. Without shepherding, the herd is on average endangered for every model and any values of the zone widths *z*_*O*_ and *z*_*A*_. Shepherding with 20 robots ensures that the herd remains entirely out of danger, for any model configuration. Deploying redundant robots is shown to not decrease performance, as the herd remains out of danger with *N*_*A*_ = 40 shepherding robots.
